# Triadic Interactions in Families of Adolescents with Anorexia Nervosa and Families of Adolescents with Internalizing Disorders

**DOI:** 10.3389/fpsyg.2016.02046

**Published:** 2017-01-05

**Authors:** Laura Balottin, Stefania Mannarini, Martina M. Mensi, Matteo Chiappedi, Michela Gatta

**Affiliations:** ^1^Interdepartmental Center for Family Research, Department of Philosophy, Sociology, Education, and Applied Psychology, Section of Applied Psychology, University of PadovaPadova, Italy; ^2^Child Neuropsychiatry Unit, Department of Brain and Behavioral Sciences, University of PaviaPavia, Italy; ^3^Child Neuropsychiatry Unit, C. Mondino National Neurological InstitutePavia, Italy; ^4^Childhood, Adolescence and Family Unit, Department of Woman’s and Child’s Health, ULSS 16 and University of PadovaPadova, Italy

**Keywords:** anorexia nervosa, family relationships, parental couple, affective regulation

## Abstract

The latest studies and practice guidelines for the treatment of adolescent patients with anorexia nervosa agree in pointing out the key role played by parents in determining the young patients’ therapeutic possibilities and outcomes. Still family functioning has usually been studied using only self-reported instruments. The aim of the present study is therefore to investigate the triadic interactions within the families of adolescents with anorexia nervosa using a semi-standardized observational tool based on a recorded play session, the Lausanne Trilogue Play (LTP). Parents and adolescent daughters, consecutively referred to adolescent neuropsychiatric services, participated in the study and underwent the observational procedure (LTP). The 20 families of adolescent girls with anorexia nervosa (restricting type) were compared with 20 families of patients with internalizing disorders (anxiety and depression). The results showed different interactive patterns in the families of adolescents with anorexia nervosa: they had greater difficulties in respecting roles during the play, maintaining the joint attention and in sharing positive affect, especially in the three-together phase (third phase). The majority of these families (12) exhibited collusive alliances. The parental subsystem appeared frequently unable to maintain a structuring role, i.e., providing help, support and guidance to the daughters, while the girls in turn often found it hard to show independent ideas and develop personal projects. Parents experienced difficulty in carving out a couple-specific relational space, from which the ill daughter was at least temporarily excluded also when they were asked to continue to interact with each other, letting the daughter be simply present in a third-part position (fourth phase). The study of the triadic interactions in the families of adolescents with anorexia nervosa may help to shift the attention from the exclusive mother–daughter relation to the involvement of the father, and of the parental couple as a whole. The family functioning is in fact well established as a maintaining factor of anorexia nervosa or vice versa as a facilitating factor in the therapeutic process.

## Introduction

### Adolescent Anorexia Nervosa and Family Relations

Anorexia nervosa is the most studied and best-known eating disorder; it usually develops during adolescence. It is likely to have a very severe prognosis, with a percentage of mortality around 1.8% in patients with onset within 18 years, that climbs to 5.9% in an unrestricted patient sample (Steinhausen, 2009). The mortality ratio increases by 5.6% per decade of illness duration (Sullivan, 1995; Herzog et al., 1996; Steinhausen, 2009). Moreover the chronicization rate is high, with a minimum of 16.9% in less severely ill patients with a relatively short illness duration (Steinhausen, 2009). Therefore the management of the early stages of the disorder’s development seems crucial in order to prevent the most negative outcomes.

Anorexia nervosa is a complex multifactorially determined eating disorder. Many different psychological, biological, environmental, familial, and sociocultural factors have been implicated in the origin and course of the disorder (Rikani et al., 2013; Lock et al., 2015). Indeed, the potential role of family factors has been increasingly recognized both in the complex pathogenic and maintenance mechanisms and in the effectiveness of the therapeutic strategies for patients with anorexia nervosa (Lyke and Matsen, 2013; Anastasiadou et al., 2014; Lock et al., 2015). Anorexia nervosa affects the adolescent and his/her family as well, seriously triggering relational difficulties. Evidence suggests that family functioning has a pivotal role in the establishment and in the evolution of the disorder (Rodríguez Martín et al., 2004; Ravi et al., 2009; Lyke and Matsen, 2013). In a cohort study (National Birth Cohort), covering a population followed from birth to the age of 30 years, Nicholls and Viner (2009) identified maternal depressive symptoms as a risk factor for the onset of anorexia nervosa. Along with depression, other psychological aspects of the patients with anorexia nervosa and of their parents were investigated, including the characteristics of the perception and imaginative elaboration of emotions and feelings (e.g., alexithymic traits) within a family, confronted by the life endangering illness of the daughter. Findings from recent studies (Balottin et al., 2014; Duclos et al., 2014) suggested in fact that not only the adolescent with anorexia nervosa but also her parents might present specific emotional difficulties within the familial relations.

Nevertheless the issue is still debated: literature data supporting the importance of the family dynamics are contradictory and limited by a wide variety of methodological approaches, mainly based on self-rated assessment (Holtom-Viesel and Allan, 2014), which implies significant limitations due to the patients’ self-awareness (Mannarini, 2009), desire and capacity to report about their own experiences and perceptions. Moreover, important authors, as Herpertz-Dahlmann et al. (2011), believe that anorexia nervosa is a predominantly neuropsychiatric disorder in its pathogenesis, which has little to do with the “model of psychosomatic family,” firstly described by Minuchin et al. (1975). The Academy of Eating Disorders (Le Grange et al., 2010) has disclosed a clear and strong position about this debated issue, declaring to be “firmly against any aetiological model of eating disorders in which family influences are seen as the primary cause of anorexia nervosa or bulimia nervosa.” From a therapeutic and family oriented point of view, it seems in fact sterile and even harmful to blame the parents, preventing them from taking the pivotal role of help and support to the adolescent care, a task for which they cannot be replaced (Novick and Novick, 2011; Apter and Palacio-Espasa, 2012). The focus of the interest therefore seems to have moved from the causal and pathogenetic role of family factors to their maintaining or vice versa facilitating role for the treatment of the disorder (Holtom-Viesel and Allan, 2014). Compared to families of daughters without an eating disorder, the families of patients with eating disorders report a more dysfunctional family functioning, although it is still unclear if there are any differences based on the different eating disorders considered (McDermott et al., 2002; Gillett et al., 2009; Holtom-Viesel and Allan, 2014). Parents of patients with eating disorders, especially of those with anorexia nervosa (Sim et al., 2009), not only experience greater family conflict but also more feelings of stress and depression. However, it is not yet known whether and how maternal perceptions differ from paternal ones (Holtom-Viesel and Allan, 2014). Moreover patients perceive their family functioning even more negatively than their parents (Dancyger et al., 2005). According to longitudinal studies (Woodside et al., 1996; Gowers and North, 1999), the patient’s more positive perception of family functioning seems to be linked to a better outcome (Holtom-Viesel and Allan, 2014).

### Why Studying Triadic Interactions? The Family Role in the Treatment of Anorexia Nervosa

The focus of the study of the family functioning seems to have moved from its potential etiologic role in maintaining the disorder to its facilitating role for the treatment of anorexia nervosa (Holtom-Viesel and Allan, 2014). The parental couple’s participation and the involvement of the whole family in the adolescent’s treatment process is recognized as a key prognostic factor by all the main guidelines for the treatment of anorexia nervosa (National Institute for Health, and Care Excellence [NIHCE], 2004; American Psychiatric Association, 2006; Hay et al., 2014; Espie and Eisler, 2015; Herpertz-Dahlmann et al., 2015; Lock et al., 2015). Some pioneering studies have also shown that encouraging paternal involvement could improve treatment outcomes (Couturier et al., 2013; Horesh et al., 2015). Yet the role of the fathers is still poorly studied and often misunderstood in favor of an exclusive and undue focus on the mother–daughter relationship.

The family therapy, which include the fathers as well as the mothers, has achieved the most relevant evidence of effectiveness (evidence type I) in the care of patients with anorexia nervosa (Fisher et al., 2010; Couturier et al., 2013) and it is considered the first-line treatment for teens under the age of 19, especially in those with recent weight loss and illness duration below 3 years (Le Grange et al., 2010; Lock, 2011; Hay et al., 2014). However, some recent studies showed that other therapies, such as adolescent-focused therapy (AFT) (Fitzpatrick et al., 2010), can also be just as effective Along with the well-established Family Based Therapy (FBT) (Le Grange and Eisler, 2009; Murray and Le Grange, 2014), other family therapeutic approaches, based on different theoretical matrixes than behavioral or systemic ones, have also shown good evidence of effectiveness: this is the case of the psychodynamic therapy based on the intra-family relationships created by the French group of the Montsouris from Paris (Godart et al., 2012). Such a therapy (added to the treatment as usual) while unlike the above-mentioned family approaches it does not tackle explicitly food-related behaviors, has proven to be effective, both in reducing the eating symptoms and in improving the general psychological functioning of the patients.

Although it is well known that different family treatments have proved to be very effective in dealing with anorexia, the functioning mechanisms of such therapies and the specific family dynamics which the treatment should more fruitfully address are still potentially fertile areas that need to be explored (Wallin and Kronvall, 2002). Observational studies of family dynamics might be the first attempt in this direction, giving the possibility to derive clinical implications, which subsequent controlled studies will support or falsify (Mann, 2003). Shedding light on dysfunctional characteristics of the family interactions might indeed be a preliminary helpful step for the promotion of the most adequate therapeutic choices for each single patient and family, at the same time directing therapeutic work on the most salient aspects that need to be modified at a family level, in order to improve the chances of the patient care. In line with these premises, in their systematic review Holtom-Viesel and Allan (2014) suggested the need to carry out further studies on family functioning in families of patients with EDs, using observer-rated clinical tools, in addition to the usual self-report.

### The Evaluation of Triadic Interactions with the Lausanne Trilogue Play

The direct observational method, based on a recorded play session, named Lausanne Trilogue Play (LTP) (Fivaz-Depeursinge and Corboz-Warnery, 1999), has already shown strong ability to detect the specific characteristics of the family triadic interactions in the context of therapeutic orientation, preventive and social interventions, in particular with infants and children (e.g., Fivaz-Depeursinge and Favez, 2006; Fivaz-Depeursinge et al., 2007, 2009; Galdiolo and Roskam, 2016). Several studies confirmed that an early evaluation of the triangular interactions can be useful to predict some core aspects of the child psychological development: children in fact learn to regulate their inner states and emotions in the context of the family relationships. Studying in a community sample the longitudinal development of the family interactions since pregnancy, Favez et al. (2012) demonstrated a relationship between the familial alliance (i.e., the quality of the triadic coordination exhibited during the video-recorded play) and the cognitive-emotional development of the child at the age of 5. Along with the child temperament, a high stable family alliance appeared to predict better outcomes in children, in particular for the development of the Theory of Mind (Baron-Cohen, 1991). Moreover a Swedish research Hedenbro and Rydelius (2014) showed that children who exhibit better triangulation capabilities within the family at 9 months have better peer and social competences at the age of 4. In turn, the early triangulation capabilities and specifically the turn-taking competence correlate with the quality of the parents’ responsiveness. The LTP may therefore be a valuable tool in identifying children and families with communication deficits and dysfunctional relational patterns at an early stage. For example, prenatal LTP may open a window into the development of the coparental behaviors and representations before the baby’s birth (Altenburger et al., 2014). A significant continuity was in fact observed between the prenatal coparental behaviors and the observed coparenting behaviors 1 year later; moreover, fathers who engaged in higher quality prenatal intuitive parental behaviors were discovered to exhibit more supportive parental behavior during the postpartum, when pregnant mothers presented lower parenting behaviors (Schoppe-Sullivan et al., 2014). The high potential for clinical intervention in relationally frail family systems was also demonstrated in a study (McHale and Coates, 2014) concerning African-American unmarried couples, which found that the majority of families showed high degrees of disengagement and tension-competitiveness, signaled by disputes and interferences.

Despite the increasing number of studies using this innovative observational tool in different family contexts (Frascarolo et al., 2005; Lavadera et al., 2011; Simonelli et al., 2012; Mazzoni et al., 2015), data regarding families with adolescent offspring are still very limited. Nevertheless a recent study (Gatta et al., 2016b), aiming to examine the usefulness of the LTP as an outcome measure, found that the LTP assessment of the family interactions might help clinicians to focus on the dysfunctional familial dynamics, thus improving the effectiveness of a video-feedback intervention with the families of children and adolescents with psychiatric disorders (i.e., significantly reducing internalizing symptoms).

These considerations pave the way to an in-depth exploration of this procedure in the context of the adolescent psychopathology, aiming to investigate if the LTP might help to discriminate core aspects of family functioning in different disorders.

### Aim and Hypotheses

The aim of the present study is to investigate the triadic interactions within the families of adolescents with anorexia nervosa, using the LTP (Fivaz-Depeursinge and Corboz-Warnery, 1999). The study aims in particular to explore:

(1) the quality of the triadic coordination, i.e., the quality of the functioning expressed by the mother–father-daughter triad while sharing the play experience and trying to reach a common goal. This measure can be a valuable index of the triadic functioning, based on the assumption that more or less functional triadic dynamics exhibited during daily life may be observed also during the videotaped procedure.
(2) The coparenting, i.e., the parental couple’s ability to play a structural role for the daughter, in particular in the third phase of the LTP procedure when the parents and the daughter are asked to interact together.
(3) The interactive dynamics within the parental couple, in particular in the fourth phase of the LTP procedure, when parents are asked to continue to interact with each other, letting the daughter in a third-part position.

To achieve these goals, we compared families of patients with anorexia nervosa with families of patients suffering from internalizing disorders (anxiety and depression) – rather than with families of adolescent girls belonging to the general population. Such a comparison allowed to highlight the characteristics of the families of daughters with anorexia nervosa, which are not simply explained by the general familial difficulties due to a daughter’s psychiatric disorder (characteristic that is present in both the family groups), regardless of the specific disorder studied.

According with the recent litterature on adolescent psychiatric disorders, the internalizing symptoms seem to coincide with a repressive, and thus maladaptive, management of affects and emotions (Rieffe and De Rooij, 2012). While externalizing and beaviural symptoms seem to indicate a more profound and resistant inability to modulate and manage emotions (Manninen et al., 2011) by means of their cognitive processing: in those cases impulsive and compulsive behaviors could be aimed at decreasing the emotional tension. Moreover major difficulties were shown to be linked to maladaptive familial characteristics, such as specific deficits in the emotional regulation within the parental couple and a neglectful parenting style perceived by the offspring (Gatta et al., 2016a).

In line with literature concerning family functioning in anorexia nervosa (Holtom-Viesel and Allan, 2014) it was hypothesized that the family interactive patterns would be different, and in particular more dysfunctional, in the case of anorexia nervosa, compared to other psychiatric disorders (i.e., internalizing disorders). Although detailed previsions were not possible, giving the explorative nature of this first study using LTP in families of patients with anorexia nervosa and families of adolescents with internalizing disorders, in general it was hypothesized that

(1) Families of patients with anorexia nervosa would display a lower triangular coordination and thus a more dysfunctional triadic functioning, compared to families of patients suffering from other emotional disorders. Moreover the triangular coordination was expected to be defective in particular during the three-together phase (third phase) of the LTP procedure due to the difficulties in enhancing and at the same time not overwhelming the daughter’s initiatives. Families of patients with internalizing disorders would display in general a more functional triangular coordination. It was hypothesized in fact that they would probably show only specific difficulties linked to the emotional contact with a depressed or anxious daughter, being neverthless able to succesfully share the game experience, although probably with more efforts than families belonging to the general population.
(2) Parents of patients with anorexia nervosa would have greater difficulties in playing a structural role for the daughter, i.e., parents would have lower scores in respecting their role, at least in the three-together phase (third phase). A specific decrease of scores in the third phase was not hypothesized in respect to the parents of the adolescents suffering from internalizing disorders, who would be able to better maintain their structural role.
(3) Parents of patients with anorexia nervosa would have greater difficulties in respecting their role also when they were asked to continue to interact with each other (fourth phase). Parents of daughters with internalizing disorders were instead not hypothesized to have major difficulties in respecting their role but only in the emotional contact, similarly to their daughters.

## Materials and Methods

### Participants

Twenty families of patients with anorexia nervosa and 20 families of adolescents with internalizing disorders (clinical comparison group) participated in the study. The adolescents had been referred consecutively (from January to June 2016) to the Childhood, Adolescence and Family Unit at the ULSS 16—University of Padova (Italy), or to the Child and Adolescent Neuropsychiatry Unit, National Neurological Institute IRCCS C. Mondino – University of Pavia (Italy).

Selection was based on the following inclusion criteria for the group with anorexia nervosa: diagnosis of anorexia nervosa, restrictive subtype according to the DSM-5 (American Psychiatric Association, 2013); female gender; age between 13 and 18 years; current BMI below the tenth percentile per age and sex. Exclusion criteria instead were: diagnosis of autism spectrum disorder, schizophrenia, learning disabilities (according to DSM-5); having received psychotherapy for a significant period (more than 3 months); insufficient understanding of the Italian language; being from a single-parent family.

Patients for the clinical comparison group with psychopathology were selected according to the following inclusion criteria: normal weight female adolescents with an anxiety (from F40.0 to F41.9) or mood disorder (depressive type, from F32.0 to F39) diagnosis (DSM-5), age between 13 and 18 years. Exclusion criteria for the families of the clinical comparison group were: presence of symptoms related to food (either restrictive or purging or binge eating conducts), diagnosis of autism spectrum disorder, schizophrenia, learning disabilities (according to DSM-5), having received psychotherapy for a significant period (more than 3 months), insufficient understanding of the Italian language, single-parent families.

The mean age of the adolescent patients with anorexia nervosa was 14.80 years (standard deviation = 1.70; range 13–18 years), and their mean current Body Mass Index was 15.55 (standard deviation = 1.34). The fathers’ mean age was 49.53 years (standard deviation = 6.16; range 36–58 years), while the mothers’ mean age was 47.00 years (standard deviation = 6.17; range 34–58 years). Concerning the clinical comparison group, it was composed of patients with Generalized Anxiety Disorder (F41.1, 8 patients), Panic Disorder (F41.0, 3 patients), Mixed Anxiety and Depressive Disorder (F41.2, 4 patients) or Persistent Depressive Disorder (Dysthymia, F34.1, 5 patients). The mean age of the adolescents was 14.85 years (standard deviation = 1.57; range 13–18 years), the fathers’ mean age was 51.11 years (standard deviation = 6.74; range 38–62 years) and the mothers’ mean age was 47.31 years (standard deviation = 6.31, range 37–58 years). The socioeconomic status (SES, Hollingshead, 1975) distributions of the two family groups (no significant differences) are presented in **Table 1**.

**Table 1:** The socioeconomic status (SES) distribution of the families of adolescents with anorexia nervosa and of the clinical comparison group families.

SES	Low	Medium low	Medium	Medium high	High
AN	2 (10%)	2 (10%)	4 (20%)	9 (45%)	3 (15%)
CTR	2 (10%)	4 (20%)	5 (25%)	4 (20%)	5 (25%)


*AN: Families of adolescents with anorexia nervosa, CTR: Clinical comparison group families.*

### Procedure

Child and adolescent psychiatrists and clinical psychologists, not directly involved in the research project, recruited parents and patients, who were consecutively referred to the services from January to June 2016, based on the diagnoses included in the study. All participants gave their written informed consent to participation in the study, within a wider project authorized by the Ethical Committee of the ULSS 16 of Padua. The study was conducted in accordance with the national and institutional code of good ethical practice.

A neuropsychiatrist with special interest and clinical experience in anorexia nervosa evaluated all patients and collected a comprehensive medical and family history. Patients, diagnosed according to the DSM-5 criteria for restricting type anorexia nervosa (American Psychiatric Association, 2013), also completed the Eating Disorder Inventory (EDI-3) (Garner, 2004). The diagnoses of anorexia nervosa and potential comorbid disorders, as well as the anxiety or depressive disorder diagnoses, were supported by the Kiddie-SADS semi-structured interview (Kaufman et al., 2000).

The families participated in a videotaped play session according to the LTP procedure, before the remaining psychological assessment. The semi-standardized observational tool, the LTP, was then scored by two specifically trained judges, blind to the results of the other tests. In rare cases of disagreement in attributing the score, an agreement score was reached through discussion.

### Measures

#### The Lausanne Trilogue Play (LTP)

The LTP is a semi-standardized observational tool based on a videotaped, semistructured play session, aimed at studying the triad as a whole as well as the organization of its parts (Fivaz-Depeursinge and Corboz-Warnery, 1999). Parents are asked to interact (i.e., imagine to organize an activity together) with their daughters in four phases. The first two phases are indicated as two-plus-one phases. In the first phase, one parent is asked to interact with the daughter, while the other parent is simply present. In the second phase, the second parent is asked to interact with the daughter, while the parent who played first is no longer involved. In the third phase (three-together phase), the parents are asked to interact together with their daughter as a triad. In the fourth phase (again a two-plus-one phase), the parents are asked to continue to interact and converse with each other, without involving the daughter. The whole play section takes approximately 15 min.

The narrative of a situation based on separation and autonomy is the theme of the triadic play, tailored for adolescent patients. Parents and daughter are asked to organize a weekend, in which the daughter will remain at home without the parents. They have to build this narrative, following the four different phases. Nevertheless in order to code the family relationships, the content of the narrative built is not very important but it is essential to note how the family shares the construction of the narrative, based on the frequency of initiatives and proposals intended to enrich the history (that should be co-constructed with the contributions of each family member).

The LTP paradigm (Fivaz-Depeursinge and Corboz-Warnery, 1999) was modified to be applied to adolescents up to 18 years (Malagoli Togliatti and Mazzoni, 2006; Lavadera et al., 2011) The coding scheme of the LTP (Malagoli Togliatti and Mazzoni, 2006) used herein was adopted and explained in other international works (Lavadera et al., 2011; Mazzoni and Lubrano Lavadera, 2013; Mazzoni et al., 2015). Fivaz-Depeursinge supervised the modifications of the task and the coding system proposed and manualized by Malagoli Togliatti and Mazzoni (2006).

The coding scheme comprises four scales, each defining an observational variable measured for each individual and for each phase and graded on a 3 point Likert scale (0 = dysfunctional, 1 = partially functional; 2 = functional). The two judges are required to define the presence – based on the frequency and duration – or the absence of some behavioral indicators (i.e., a given behavior during the activity) in order to assign a score to each functional level: participation, organization, shared attention, and emotional contact.

Participation is the ability to get involved in the same interactive space (i.e., the disposition to take part in the interaction, expressed also in the body orientation), getting in touch with the other family members (McHale et al., 2001). It indicates the inclusion or the exclusion of a member of the triad and is assessed by observing how the participant places his body in the interactive field, in particular, if he/she sits correctly and if he/she orients his/her body to other family members and the task. Participation represents the simplest function to be achieved and the lowest step to establish collaboration, which have to remain stable throughout the interaction time, because without an adequate physical orientation and closeness it is not possible for the participant to interact and play with the other family members.

Organization is the capacity of each participant to play a role coherent with the different parts of the play: the roles played by each parent and the daughter have to be different according to the different phases. When a parent is in the active position, he/she should help his/her daughter in the activity providing suggestions and supporting and encouraging the girl’s proposals. When a participant is in the observer position, he/she should take up this role quickly, remain involved in the interaction, demonstrating his/her support for the partner without trying to replace him/her, and repairing any errors or deficiencies of the other parent. In the first two parts, the daughter should work together with the active parent, proposing initiatives but also being able to be guided to the target. In the third part, the three members should coordinate and alternate without competing with each other, proposing initiatives that facilitate the inclusion of all members in the current play. In the last phase, the parents must be able to leave a certain autonomy of action to their daughter and to interact with each other as a couple; the daughter must be able to accept this exclusion and continue the assigned task alone. This functional level (organization) evaluates also the participants’ coherence to the subsystem to which they belong: since parents represent the structuring system and the daughter the evolutive subsystem, they are expected to play differently.

Shared attention assesses the ability to reach and maintain a joint attentive focus, shared by the triad during the play, which allows to communicate meanings and affections and to co-construct a common narrative plot during the whole play. The shared attention is achieved when each of the participants (regardless of the role played in a specific phase) pays attention to the interactive elements, to the ongoing activities and to the actions of the other participants, sharing meanings with each other by mean of looks, gestures, and words. A participant gets a functional score if he/she watches the game and the other participants, speaks about the game and its plot, follows the play goal agreeing with the other family members. The coherence among gazes, actions and verbalization is crucial in achieving a functional focalization.

The emotional contact is the more complex functional level, which implies the emotional sharing, the reciprocity and communion of affections (McHale et al., 2001). It is evaluated observing the affective tone shown during the triadic play by means of ways of looking, physical contact, verbal communication of affections and reinforcements within the triad. It is expected that the individual smiles spontaneously, makes jokes and laughs at others’ jokes, he/her should may express approval and praise for the initiatives proposed, showing complicity with the partner. In short, if each participant is able to have fun in an authentic and shared way, a good affective contact is achieved.

This coding system allows having a score for each family member and for each functional level in each part of the play, a combined family score for each functional level and for each part and finally a global score of family functioning. The global score of the family is the sum of the overall scores for each family member and represents a dimensional assessment of the triadic coordination that ranges from 0 (absent coordination) to 40 (maximum coordination). The cut-off to distinguish the families into categories of different alliances are: 0 to 23 for low coordination families and 24 to 40 for those families with a high coordination. According to the Lausanne Group, the family alliance construct indicates the quality of the coordination expressed by the family while sharing the play experience and while trying to reach a common goal. In particular four types of family alliances were distinguished based on the global coordination exhibited:

- Disturbed (global score 0–16): the family cannot carry out the task of the play because the roles are not well defined and undergo continuous interference. The game pieces are confused and overlapping, generating a continuous tension and ambiguity or even the exclusion of a member of the triad. The emotional climate is negative, although it may present a “false-positivity” (positivity surface expressions which mask deep underlying negative emotions);
- Collusive (global score 17–23): there is a division of the parental subunit; these families fail to achieve the goal of the game and to share fun. The difficulty of the parental subsystem to provide help and guidance to the child or of the child to accept the “guidance” of the parents is clear. Competition between parents is present and can become evident. The emotional climate is crossed by a constant tension repaired and often hidden by an apparent serenity;
- in tension (global score 24–32): the family plays together, but encounters obstacles that create “tension” in the emotional climate, otherwise serene. The coparenting coordination in some moments of the game is lost, threatening the emotional sharing. Despite these ups and downs the parents try to repair the wrong co-ordinations, restoring a cooperative atmosphere;
- collaborative (global score 33–40): it is observed in families that show a good level of cooperation and coordination. The family plays together as a team, reaching the established goal of affective sharing. Parents work together and coordinate to facilitate the child; in the case of “missteps,” the parental subsystem manages to repair effectively.

The inter-rater reliability between the two judges’ scores was evaluated for the overall score and for every single dimension, using Cohen’s K coefficient. Although the agreement was sometimes lower for single individuals in specific phases, the overall reliability estimates proved to be much higher than the recommended threshold value of 0.60 concerning the total and the four triadic functional levels, ranging from 0.70 to 1. As regards the alliances categories, the agreement between the two judges reached the score of 1, indicating a perfect agreement.

### Analyses

A log-linear model approach for categorical variables was applied in order to analyze the families of adolescents with anorexia nervosa and compare them with the clinical control ones (Agresti, 2002). Modeling the data on the basis of the log-linear analysis allows estimating parameters that describe and evidence the significant interactions between variables measured by the LTP and the two groups of families, the research group with anorexia nervosa (AN), and the clinical comparison group with anxiety or depression (CTR). Namely the log-linear analysis is applied on data cross classified by means of contingency tables, which in this study are bivariate tables, where variable one (var 1) is represented by the categories of the LTP variables and variable two (var 2) corresponds to the family variable, distinguished in two groups, AN and CTR. For each cell of the table which represents the interaction of each category of var 1 with each of the two groups (var 2), a parameter is estimated which describes the strength of the association. Statistically significant (*p* < 0.05) positive or negative estimates are interpretable and can be discussed. Similarly in this study the log-linear analysis is used to study the interactions between LTP variables categories and each family role (i.e., father, mother, and daughter), which belongs to the two groups, that is, for instance, the LTP categories are cross classified with two the groups, AN-Father vs. CTR-Father; the same for mother and daughter. The use of log-linear models for categorical data in psychological research has been consistently used in existing literature (e.g., Mannarini and Boffo, 2015; Capaldi et al., 2016; Mannarini et al., 2016b).

Analyses were performed by means of bidimensional contingency tables; interaction parameters and standard errors were estimated in order to calculate a standard score for each interaction. Estimated parameters are presented in **Tables 2**–**5**, with statistically significant values evidenced by the corresponding probability errors (^∗^*p* < 0.05, ^∗∗^*p* < 0.01, ^∗∗∗^*p* < 0.001, tendency 0.05 < *p* < 0.07).

**Table 2:** Log-linear estimated parameters to compare the research and clinical control families in relation to four Alliances Categories (disturbed, collusive, in tension, and collaborative).

Alliances category	AN	CTR
Disturbed	0.63	-0.63
Collusive	1.44^∗∗^	-1.44^∗∗^
In tension	-0.54°	0.54°
Collaborative	-1.53^∗∗^	1.53^∗∗^


*AN: Families of adolescents with anorexia nervosa, CTR: Clinical comparison group families. °0.05 < *p* < 0.07, ^∗∗^*p* < 0.01.*

**Table 3:** Organization: Log-linear estimated parameters to compare the research and clinical comparison groups (whole family and family members’ contribution) in relation to three family functioning categories in phases 3 and 4 of the play.

*Phase 3*	AN	CTR	AN-F	CTR-F	AN-M	CTR-M	AN-D	CTR-D
Dysfunctional	1.07**	-1.07**	0.71	-0.71	0.83*	-0.83*	0.69	-0.69
Partially functional	-0.02	0.02	0.18	-0.18	0.18	-0.18	-0.17	0.17
Functional	-1.05**	1.05**	-0.89**	0.89**	-1.01**	1.01**	-0.52*	0.52*
*Phase 4*	AN	CTR	AN-F	CTR-F	AN-M	CTR-M	AN-D	CTR-D
Dysfunctional	0.92**	-0.92**	1.09**	-1.09**	1.06**	-1.06**	0.62*	-0.62*
Partially functional	-0.38	0.38	-0.32	0.32	-0.34	0.34	-0.13	0.13
Functional	-0.54*	0.54*	-0.77**	0.77**	-0.72**	0.72**	-0.49*	0.49*


*AN: Families of adolescents with anorexia nervosa, CTR: Clinical comparison group families. AN-F, Anorexia nervosa-Father; CTR-F, Clincal control - Father; AN-M, Anorexia nervosa -Mother; CTR-M, Clinical control -Mother; AN-D, Anorexia nervosa-Daughter; CTR-D, Clincal control -Daughter. Phase 3– Parents and daughter interact as a triad. Phase 4– Parents interact and converse with each other, without involving the daughter ^∗^*p* < 0.05, ^∗∗^*p* < 0.01.*

**Table 4:** Shared attention: Log-linear estimated parameters to compare the research and clinical comparison groups (whole family and family members’ contribution) in relation to three family functioning categories in phases 1 and 3 of the play.

*Phase 1*	AN	CTR	AN-F	CTR-F	AN-M	CTR-M	AN-D	CTR-D
Dysfunctional	0.44	-0.44	0.32	-0.32	-0.02	0.02	0.01	-0.01
Partially functional	0.09	-0.09	0.14	-0.14	0.47	-0.47	0.18	-0.18
Functional	-0.53^∗^	0.53^∗^	-0.46°	0.46°	-0.45	0.45	-0.19	0.19
*Phase 3*	AN	CTR	AN-F	CTR-F	AN-M	CTR-M	AN-D	CTR-D
Dysfunctional	0.72°	-0.72°	0.56	-0.56	0.57	-0.57	0.71	-0.71
Partially functional	-0.25	0.25	0.08	-0.08	0.02	-0.02	0.16	-0.16
Functional	-0.47°	0.47°	-0.64^∗^	0.64^∗^	-0.59^∗^	0.59^∗^	-0.55^∗^	0.55^∗^


*AN: Families of adolescents with anorexia nervosa, CTR: Clinical comparison group families. AN-F, Anorexia nervosa-Father; CTR-F, Clinical control - Father; AN-M, Anorexia nervosa -Mother; CTR-M, Clinical control -Mother; AN-D, Anorexia nervosa-Daughter; CTR-D, Clinical control -Daughter.*

*Phase 1–One parent is asked to interact with the daughter, while the other parent is simply present.*

*Phase 3–Parents and daughter interact as a triad.*

*°0.05 < *p* < 0.07, ^∗^*p* < 0.05.*

**Table 5:** Affective contact: Log-linear estimated parameters to compare the research and clinical comparison groups (whole family and family members’ contribution) in relation to three family functioning categories in phases 3 and 4 of the play.

*Phase 3*	AN	CTR	AN-F	CTR-F	AN-M	CTR-M	AN-D	CTR-D
Dysfunctional	0.98*	-0.98*	0.65	-0.65	0.63	-0.63	0.81	-0.81
Partially functional	-0.02	0.02	-0.05	0.05	-0.14	0.14	-0.10	0.10
Functional	-0.96*	0.96*	-0.60*	0.60*	-0.49	-0.49	-0.71*	0.71*
*Phase 4*	AN	CTR	AN-F	CTR-F	AN-M	CTR-M	AN-D	CTR-D
Dysfunctional	1.14*	-1.14*	0.92*	-0.92*	0.94*	-0.94*	0.73	-0.73
Partially functional	-0.11	0.11	-0.07	0.07	0.33	-0.33	-0.27	0.27
Functional	-1.03*	1.03*	-0.85**	0.85**	-1.27**	1.27**	-0.46	0.46


*AN: Families of adolescents with anorexia nervosa, CTR: Clinical comparison group families. AN-F, Anorexia nervosa-Father; CTR-F, Clinical control - Father; AN-M, Anorexia nervosa -Mother; CTR-M, Clinical control -Mother; AN-D, Anorexia nervosa-Daughter; CTR-D, Clinical control -Daughter. Phase 3– Parents and daughter interact as a triad. Phase 4–Parents interact and converse with each other, without involving the daughter. ^∗^*p* < 0.05, ^∗∗^*p* < 0.01.*

In order to evaluate the quality of the coordination expressed by the families during the play, the four alliance categories (namely disturbed, collusive, in tension, collaborative) were analyzed comparing the two family groups (**Table 2**). For each play functional level (participation, organization, shared attention, affective contact), and for each part of the play (phases 1, 2, 3, and 4), the three categories of interactive functioning (dysfunctional, partially functional and functional) were analyzed in relation to the two family groups (AN, CTR) (**Tables 3**–**5**).

## Results

### The Family Global Coordination

Considering the Alliances categories (**Table 2**), the families of adolescents with anorexia nervosa seem to present collusive alliances significantly more than the clinical control families during the LTP play; on the other hand, a high coordination (corresponding to the “in tension” alliances and the collaborative alliances) was more frequently seen in the clinical control families. In synthesis, the overall triadic coordination exhibited by the families of adolescents with anorexia nervosa was significantly lower than that of the families from the clinical comparison group. The low coordination alliances, in particular the collusive ones, were characteristic of the families of patients with anorexia nervosa, that were mostly judged to have dysfunctional family alliances. Vice versa the two alliances included in the functional polarity, i.e., the cooperative and in tension ones, were significantly more represented in the families of patients with emotional symptoms (depression or anxiety).

### The Organization of the Roles

**Table 3** provides results concerning the Organization functional level within the family; significant results were found as regard *Phases 3* and *4.* In *Phase 3* families of adolescents with anorexia nervosa were evaluated more often in the dysfunctional category, evidencing the problematic interaction within the triad; conversely the clinical control families were associated more often with the functional category, showing that in these families the organization of the roles within the triad is more coherent. More accurate analyses regarding the role of each member in the families evidence that all the three members (father, mother, and daughter) are better organized while interacting within the triad in the clinical control families. Like in *Phase 3*, the control parents seem to be able to positively interact with each other, also when the daughter is not involved in the conversation (*Phase 4*); their interactive dynamic is in fact maintained at a functional level, whereas the interaction of the parents of the daughter with anorexia nervosa becomes dysfunctional.

The triads from the families of girls with anorexia nervosa therefore did not comply with their role in the third and in the fourth phase. All members of the family had difficulties in establishing triangular interaction, failing to coordinate with each other during the third phase. Moreover the daughters with anorexia nervosa as well as their mothers and fathers scored lower in the organization of the roles, not only in the third but also in the fourth phase.

### The Shared Attention within the Triad

Considering the Shared attention functional level, significant results are observed in Phases 1 and 3 of the play (see **Table 4**). In these phases, appropriate joint attention is achieved more often by the clinical control families: as to Phase 3, the contribution of each member of the clinical control family is relevant; as to phase 1, we observed only a tendency toward a significant positive association between daughters of the clinical comparison group and a functional category of shared attention.

In summary, shared attention was lower in families of daughter with anorexia nervosa in the first as well as in the third phase, because of the specific difficulty in maintaining a triangulation and in including a third individual in it. In the first phase (mother–daughter dyadic interaction, father as participant observer) the fathers of patients with anorexia nervosa obtained a significantly lower score in the shared attention, while the scores achieved by the mothers and the daughters were substantially comparable to those of the clinical comparison group. In this first phase in fact the father’s contribution was predominant in producing the attentional dysfunction of the whole family since the father did not pay any attention to what was happening between the mother and the daughter during their dyadic interaction and isolated himself. Whereas in the third phase of the LTP play, all the three components of the families of patients with anorexia nervosa contributed equally to the attentive dysfunctions as it was very difficult to achieve the co-construction of a shared narrative plot in the three-together phase.

### The Emotional Contact

**Table 5** shows the estimated values of the log-linear analysis between the two family groups and the functional levels of the Emotional contact regarding Phases 3 and 4. As far as emotional contact is concerned for both phases, families of adolescents with anorexia nervosa seem to be more dysfunctional; in particular in phase 3, emotional sharing and affective reciprocity seem to be more appropriate for the clinical control fathers and daughters; in phase 4, the affective contact of fathers and mothers belonging to the research group is significantly more dysfunctional.

In general the families of patients with anorexia nervosa have greater difficulties in sharing positive emotions both in the third and in the fourth phases. In the third phase the difficulty was prevalently on the fathers’ and on the daughters’ part: unlike the mothers, they scored in fact lower than the controls in the emotional contact. While during the fourth phase it is the parental couple to have more difficulties in managing the task of the play and sharing emotions.

## Discussion

### The Family Global Coordination

Comparing families of patients with anorexia nervosa with families of adolescents suffering from internalizing disorders (anxiety and depression) helped highlighting some dysfunctional interactive patterns, which were more evident in the former families of patients. In line with our first hypothesis, in general the families of girls with anorexia nervosa, often categorized as collusive during the LTP play, failed to achieve the goal of the play and to share fun and pleasure. The collusive alliances are characterized by great difficulty on the part of the parental subsystem in providing help and guidance to the child and/or on the part of the child in accepting the parents’ guidance (Malagoli Togliatti and Mazzoni, 2006). Along with the overall family coordination (expressed by the alliance category), the LTP functional levels of organization, shared attention and emotional contact were poorer in families of adolescents with anorexia nervosa during some specific phases of the play. More dysfunctional triadic patterns were found in families of patients with anorexia nervosa specifically in respect to the third (three-together) and the fourth (parents) phases, with the only exception of a difference in the first phase concerning the shared attention. It was in fact hypothesized that the three-together phase (the third) might elicit more difficulties in the families of adolescents with anorexia nervosa, due to the parents’ difficulties in supporting together the daughter without being too intrusive, given her life-threatening condition.

### The Organization of the Roles

If the participation in the play was somehow guaranteed to every participant within the families of patients with anorexia nervosa as well as within the families of patients suffering from other disorders, the organization of roles was instead significantly deficient in the former families. Not only parents of patients with anorexia nervosa didn’t manage to respect their role during the third and the fourth phases, in line with our second and third hypotheses, but also, differently from what hypothesized, the daughters had significantly more difficulties to cope with their role than adolescents with internalizing disorders. Although the attempts of the daughters with anorexia nervosa to include both parents and to establish an alternation between them were sometimes observable, the division within the parental couple made often it impossible for parents as well as for the daughters to respect each other’s initiatives and to achieve a triangulation during the three-together phase. Moreover the daughter was often included in the interaction, also when the parents were asked to continue to interact with each other, letting the daughter be simply present in a third-part position (fourth phase). Similarly to our results, different studies, conducted using the LTP (Fivaz-Depeursinge and Favez, 2006; Fivaz-Depeursinge et al., 2007, 2009), have shown that sometimes children need to use their triangular and communicative abilities with the purpose of relieving the tension within the conflicting parental couple, being therefore entangled with the couple dinamics. With the necessary caution in comparing children with adolescents, this reference to the infant age might suggest that in some cases the daughter with anorexia nervosa was making the task of managing the relationship between the parents. Nevertheless, it is at the same time undoubtedly true that the adolescent’s life-threatening condition may represent a traumatic factor for the parents, that often seem to be compelled to focus exclusively on the daughter’s condition, in a desperate attempt to avert the worst (Balottin et al., 2014). Moreover, besides being a life-threatening condition, anorexia nervosa is also a chronic illness characterized by the resistance on the part of the adolescent to take part in a therapeutic process. Thus the constant involvement of the parents in the treatment is often necessary in order to maintain the adolescent’s adherence to the therapeutic plan.

### The Shared Attention within the Triad

Shared attention was more compromised in families of patients with anorexia nervosa since it was very difficult to achieve the co-construction of a shared narrative plot, which was instead interrupted and restarted every time that a new phase started. Some families for example interpreted the goal of the play as if participants were asked to organize two different weekends, the mother’s one and the father’s one, and the daughter was called to choose the best in the third phase. In these cases the fourth phase was usually not even carried out, showing impossibility on the parents’ part to carve out a couple-specific relational space. This can be easily understood bearing in mind the daughter’s alarming and life-threatening condition, which is likely to completely absorb the parents’ attentions. Moreover the interaction was often maintained at a dual level, where the third part is excluded from the attentional space. In the majority of the cases this implies that the mother and the daughter did not pay attention to the father when he was in the role of participant observer, as if he did not exist in the interactive space, in particular in the first phase (mother–daughter, father as participant observer). The father in turn did not pay any attention to what was happening in the interaction, undertaking different lonely activities (i.e., writing messages, answering the phone, checking the agenda etc.). This finding points to a major role for fathers in the complex relational dynamics of the triad and of the whole family of the patients with anorexia nervosa. The exclusion of the father may in fact represent a risk factor for the daughter’s psychopathology while his participation may be considered a protective factor in the adolescent’s development, in line with the studies that indicate that the paternal involvement in the child care produces well-being with permanent effects (Gottman, 1997; Lamb, 2010). Although the role of the fathers is often unrecognized in favor of an exclusive concentration on the relationship of the mother and her daughter, some pioneering findings have shown that the paternal involvement in the treatment is crucial in gaining a positive outcome in anorexia nervosa (Godart et al., 2012; Couturier et al., 2013; Hay et al., 2014; Horesh et al., 2015). On the contrary a defensive and fleeing paternal response to the daughter’s severe pathology can trigger a vicious circle and therefor probably prejudice the patient’s development. This paternal tendency may therefore be a crucial aspect to be targeted and modified within a treatment designed for the families of patients with anorexia nervosa, since fostering paternal warmth and participation may improve the family relations (Godart et al., 2012; Duclos et al., 2014).

### The Emotional Contact

Differently from what hypothesized, in the third and in the fourth phases, a constant tension, unrepaired, traversed the affective climate to a greater extent in the families of daughters with anorexia nervosa. The father-daughter couple appeared to be oriented on a similar tepid affective polarity, while the mother often expressed feelings more directly and maintained different emotional characteristics, as shown in a previous study (Balottin et al., 2014). During the third phase of the LTP in fact the affective quality of the interaction was compromised by the father’s and the daughter’s difficulty to keep in touch with each other and spontaneously share affects. This is in line with the findings of other studies conducted on different samples of female adolescents with anorexia nervosa, where fathers were shown to have a pivotal role in the potential emotional deficits of families with daughter with anorexia nervosa (Balottin et al., 2014; Duclos et al., 2014). The paternal emotional absence or coldness (for example in the case of an alexithymic father) may represent a defensive reaction in front of the daughter’s illness but it in turn entails less affective connection and consequently may affect the quality of family interactions and the daughter’s outcome, which need to be avoided.

Parents showed high degrees of tension, competitiveness, and conflicts, which often made it impossible for them to speak with each other, without the daughter’s intervention, and sometimes, even, perform the fourth phase. The parental conflict, manifested in these specific difficult circumstances, seems to negatively impact not only on the partners but on the family and the daughters as well. In general, concerning the affective quality, a potentially strong continuity between the couple and the triadic relation, including the offspring, has been shown (Kitzmann, 2000). It is not only the influence of each parent separately that would provide a matrix for the daughter’s emotional development but also, and even more significantly, the affective regulation of the parental couple, as expressed in the complex relational dynamics of the triad (Kooiman et al., 2004; Gatta et al., 2016a). Evaluating the couple relationship, starting from the conflicts and distress, can therefore prove to be useful in order to better understand the functioning of the whole family and therefore plan treatments, tailored for the patient as well as for the family needs (Gatta et al., 2009, 2011; Mannarini et al., 2016a). A relationship in which emotions and thoughts are shared within the attachment relationship (Mannarini and Boffo, 2014) with the father as well as with the mother is able to facilitate the development of an adaptive affective regulation and a rich inner emotional life in the offspring (Taylor et al., 1997), thus enhancing the possibility of recovery of the daughter with anorexia nervosa.

### Limitations of the Study and Future Developments

Comparing families of patients with anorexia nervosa with families of patients suffering from other emotional disorders (anxiety and depression), rather than families with adolescent girls belonging to the general population, helped highlighting the former families’ characteristics, which are not simply explained by the general familial difficulties due to a daughter’s psychiatric disorder, regardless of the specific disorder studied. However, one of the major limitations of the study lies in the difference between the two family groups studied, although they belong to the same population of patients referred to the services: the clinical comparison group with psychopathology was in fact selected as convenience sample in order to be sampled in the same way as the research group (Mann, 2003). Nevertheless the two groups are different not only concerning the respective disorder investigated but probably also for what concerns the perceived severity of the disorder, especially for what concerns the physical consequences. Anorexia nervosa is a far more invalidating disorder than depression and anxiety, having a higher rate of life-endangering physical symptoms, not to mention that unlike the family of individuals with depression and anxiety, the family of individuals with anorexia nervosa has to deal with the more intense resistance of the adolescent to adhere to a therapeutic treatment and to accept the parental involvement. Subsequent studies may partially overcome this limitation by exploring the potential influence of the subjective severity of the respective disorder, as evaluated by each family member, on the triadic family functioning. Moreover girls with anorexia nervosa can have comorbidities including anxiety and/or depression. In our sample, an additional diagnosis was done in seven patients: Separation Anxiety Disorder (two patients), Major Depressive Disorder (two patients), Persistent Depressive Disorder (three patients). This was a plausible confounding factor, but we decided not to exclude these patients because this kind of comorbidities has been repeatedly described in scientific literature as “highly frequent” in girls with anorexia nervosa (Swanson et al., 2011).

To end with, the characteristics belonging to families of adolescents with anorexia nervosa might be better clarified by using more than a unique one clinical comparison group in subsequent studies. In addition to the clinical comparison groups formed by families of adolescents with a different psychiatric disorders, future studies may include a non-clinical comparison group of families of adolescents from the general population and a clinical comparison group comprising families of daughters with a body physical syndrome linked to a medical condition (e.g., functional hypothalamic amenorrhea or medical gastrointestinal disorder). This might help to shed some light on the characteristics linked prevalently to the daughters’ physical symptoms in respect to the possible specific psychological and relational characteristics of the patients with anorexia nervosa and their parents, displayed during the play interaction.

### Clinical Implications

Potential clinical implications can be drawn from the picture of the triadic relations interactions, with respect to the key role played by parents, and in particular by fathers, in the families of adolescents with restricting type anorexia. The findings of the present study suggest in fact that the collusive families of patients with anorexia nervosa often exhibit a division within the parental subunit, along with a competitive climate between parents, which can be either manifested or hidden by an apparent serenity (Malagoli Togliatti and Mazzoni, 2006). In particular within the triads of the research group, the quality of interaction showed a deterioration in the third (three-together) phase where families were required to display a greater triangular coordination. The parental subsystem experienced difficulties in maintaining a structuring role in relation to the daughter’s initiatives, providing her help, support, and guidance; adolescents with anorexia nervosa in turn struggled in showing independent proposals and in developing personal projects and ideas. Moreover the fourth phase of the LTP showed major difficulties on the parents’ part to carve out a couple-specific relational space.

Since the most recent literature emphasizes that the involvement of the parental couple and of the whole family cannot be disregarded in treating adolescents with eating disorders (National Institute for Health, and Care Excellence [NIHCE], 2004; American Psychiatric Association, 2006; Hay et al., 2014; Espie and Eisler, 2015; Herpertz-Dahlmann et al., 2015; Lock et al., 2015), the potentially dysfunctional interactive dynamics within the parental couple and within the triad emerged from the study may represent an obstacle for an effective treatment. In line with the current literature (Godart et al., 2012; Duclos et al., 2014), the results of our study might therefore support the clinical indication to tackle the emotional contact and to improve the father’s participation in the mother–daughter relationship. Paternal involvement and warmth proves in fact to be fundamental for the outcome and those fathers who tend to slip away and remain excluded emotionally and concretely need to be encouraged and supported (Godart et al., 2012; Couturier et al., 2013; Hay et al., 2014; Horesh et al., 2015).

Evaluating if it is possible to constructively involve the family in the treatment is indeed a mandatory step in order to avoid a failure of the family treatment, caused primarily by an inappropriate or premature therapeutic choice (Espie and Eisler, 2015; Herpertz-Dahlmann et al., 2015). Treatments often need to be tailored not only to the patient but also to each family’s needs, both in the case of anxiety or depression and in the case of anorexia nervosa (Diamond-Raab and Orrell-Valente, 2002; Gatta et al., 2010, 2014; Mannarini et al., 2013). Although being the therapeutic first choice in adolescent anorexia nervosa, the familial approach was demonstrated to be not always effective, especially in the cases where it is not possible to constructively involve the family in the treatment (Espie and Eisler, 2015; Herpertz-Dahlmann et al., 2015). On the other hand if the family relationships are too dysfunctional, the individual psychotherapy can achieve only partial results. Subsequent studies may explore if the LTP observational procedure, shedding light on the potentially dysfunctional interactive dynamics, might be helpful in assessing whether, in a specific moment, it is possible to constructively engage the family in the treatment of the patient with anorexia nervosa. This study represents in fact an initial step in the direct observation of family interactions in anorexia nervosa, which may only offer some preliminary results, to be further enriched, confirmed or disconfirmed.

## Ethics Statement

Ethical Committee of the ULSS 16 of Padua, Italy (Deliberation 871, 10/10/2013) Patients and parents have given their written consent to the participation in the study in accordance with the national and institutional code of good ethical practice and with the Declaration of Helsinki.

## Author Contributions

LB, SM, and MC wrote the manuscript. MM and MG revised it critically. LB, SM, MM, MC, and MG contributed to study design, data discussion and critical evaluation of the manuscript. LB and MM contributed to data collection. SM preformed analyses and contributed to data interpretation. All the authors approved the final manuscript and agreed to be accountable for all the aspects of the study.

## Conflict of Interest Statement

The authors declare that the research was conducted in the absence of any commercial or financial relationships that could be construed as a potential conflict of interest.

## References

[B1] Agresti A. (2002). *Categorical Data Analysis.* New York, NY: Wiley.

[B2] Altenburger L. E. Schoppe-Sullivan S. J. Lang S. N. Bower D. J. Kamp Dush C. M. (2014). Associations between prenatal coparenting behavior and observed coparenting behavior at 9-months postpartum. *J. Fam. Psychol.* 28 495–504. 10.1037/fam0000012 25000135 PMC4485399

[B3] American Psychiatric Association (2006). *American Psychiatric Association Practice Guidelines for the Treatment of Psychiatric Disorders: Compendium 2006.* Arlington, VA: American Psychiatric Association.

[B4] American Psychiatric Association. (2013). *Diagnostic and Statistical Manual of Mental Disorders*, 5th Edn Arlington, VA: American Psychiatric Association.

[B5] Anastasiadou D. Medina-Pradas C. Sepulveda A. R. Treasure J. (2014). A systematic review of family caregiving in eating disorders. *Eat. Behav.* 15 464–477. 10.1016/j.eatbeh.2014.06.001 25064301

[B6] Apter G. Palacio-Espasa F. (2012). Parentalité coupable ou hypercoupable? Telle est la question! [Parenthood, guilty or very guilty? This is the question]. *Enfances Psy* 57 36–46. 10.3917/ep.057.0036

[B7] Balottin L. Nacinovich R. Bomba M. Mannarini S. (2014). Alexithymia in parents and adolescent anorexic daughters: comparing the responses to TSIA and TAS-20 scales. *Neuropsychiatr. Dis. Treat.* 10 1941–1951. 10.2147/NDT.S67642 25336959 PMC4200172

[B8] Baron-Cohen S. (1991). “Precursors to a theory of mind: understanding attention in others,” in *Natural Theories of Mind: Evolution, Development and Simulation of Everyday Mindreading*, ed. Whiten A. (Oxford: Blackwell), 233–251.

[B9] Capaldi S. Asnaani A. Zandberg L. J. Carpenter J. K. Foa E. B. (2016). Therapeutic alliance during prolonged exposure versus client-centered therapy for adolescent posttraumatic stress disorder. *J. Clin. Psychol.* 72 1026–1036. 10.1002/jclp.22303 27105016

[B10] Couturier J. Kimber M. Szatmari P. (2013). Efficacy of family-based treatment for adolescents with eating disorders: a systematic review and meta-analysis. *Int. J. Eat. Disord.* 46 3–11. 10.1002/eat.22042 22821753

[B11] Dancyger I. Fornari V. Scionti L. Wisotsky W. Sunday S. (2005). Do daughters with eating disorders agree with their parents’ perception of family functioning? *Compr. Psychiatry* 46 135–139. 10.4236/ojn.2012.24059 15723031

[B12] Diamond-Raab L. Orrell-Valente J. K. (2002). Art therapy, psychodrama, and verbal therapy. An integrative model of group therapy in the treatment of adolescents with anorexia nervosa and bulimia nervosa. *Child Adolesc. Psychiatr. Clin. N. Am.* 11 343–364. 10.1016/S1056-4993(01)00008-6 12109325

[B13] Duclos J. Dorard G. Berthoz S. Curt F. Faucher S. Falissard B. (2014). Expressed emotion in anorexia nervosa: what is inside the ‘black box’? *Compr. Psychiatry* 55 71–79. 10.1016/j.comppsych.2013.10.002 24199888

[B14] Espie J. Eisler I. (2015). Focus on anorexia nervosa: modern psychological treatment and guidelines for the adolescent patient. *Adolesc. Health Med. Ther.* 6 9–16. 10.2147/AHMT.S70300 25678834 PMC4316908

[B15] Favez N. Lopes F. Bernard M. Frascarolo F. Lavanchy Scaiola C. Corboz-Warnery A. (2012). The development of family alliance from pregnancy to toddlerhood and child outcomes at 5 years. *Fam. Process* 51 542–556. 10.1111/j.1545-5300.2012.01419.x 23230984

[B16] Fisher C. A. Hetrick S. E. Rushford N. (2010). Family therapy for anorexia nervosa. *Cochrane Database Syst. Rev.* 4:CD004780 10.1002/14651858.CD004780.pub2 20393940

[B17] Fitzpatrick K. K. Moye A. Hoste R. Lock J. le Grange D. (2010). Adolescent focused psychotherapy for adolescents with anorexia nervosa. *J. Contemp. Psychother.* 40 31–39. 10.1007/s10879-009-9123-7

[B18] Fivaz-Depeursinge E. Corboz-Warnery A. (1999). *The Primary Triangle: A Developmental Systems View of Mothers, Fathers, and Infants.* New York, NY: Basic Books.

[B19] Fivaz-Depeursinge E. Favez N. (2006). Exploring triangulation in infancy: two contrasted cases. *Fam. Process* 45 3–18. 10.1111/j.1545-5300.2006.00077.x 16615250

[B20] Fivaz-Depeursinge E. Frascarolo F. Lopes F. Dimitrova N. Favez N. (2007). Parents-child role reversal in trilogue play: case studies of trajectories from pregnancy to toddlerhood. *Attach. Hum. Dev.* 9 17–31. 10.1080/14616730601151425 17364480

[B21] Fivaz-Depeursinge E. Lopes F. Python M. Favez N. (2009). Coparenting and toddler’s interactive styles in family coalitions. *Fam. Process* 48 500–516. 10.1111/j.1545-5300.2009.01298.x 19930435

[B22] Frascarolo F. Zaouche-Gaudron C. Rouyer V. Favez N. (2005). Variations in fathers’ discourse on fatherhood and in family alliances during infancy. *Eur. J. Psychol. Educ.* 20 185–199. 10.1007/BF03173507

[B23] Galdiolo S. Roskam I. (2016). From me to us: the construction of family alliance. *Infant Ment. Health J.* 37 29–44. 10.1002/imhj.21543 26715070

[B24] Garner D. M. (2004). *Eating Disorder Inventory-3. Professional Manual.* Lutz, FL: Psychological Assessment Resources.

[B25] Gatta M. Balottin L. Mannarini M. Chesani G. Del Col L. Spoto A. (2016a). Familial factors relating to alexithymic traits in adolescents with psychiatric disorders. *Clin. Psychol.* 10.1111/cp.12098

[B26] Gatta M. Sisti M. Sudati L. Miscioscia M. Simonelli A. (2016b). The Lausanne trilogue play within the outcome evaluation in infant mental health: a preliminary report. *Res. Psychother. Psychopathol.* 19 19–30. 10.4081/ripppo.2016.198

[B27] Gatta M. Dal Zotto L. Del Col L. Spoto A. Testa C. P. Ceranto G. (2010). Analytical psychodrama with adolescent suffering from psycho-behavioral disorder: short-term effects on psychiatric symptoms. *Arts Psychother.* 37 240–247. 10.1016/j.aip.2010.04.010

[B28] Gatta M. Dal Zotto L. Nequinio G. Del Col L. Sorgato R. Ceranto G. (2011). Parents of adolescents with mental disorders: improving their caregiving experience. *J. Child Fam. Stud.* 20 478–490. 10.1007/s10826-010-9415-2

[B29] Gatta M. Gallo C. Vianello M. (2014). Art therapy groups for adolescents with personality disorders. *Arts Psychother.* 41 1–6. 10.1016/j.aip.2013.11.001

[B30] Gatta M. Ramaglioni E. Lai J. Svanellini L. Toldo I. Del Col L. (2009). Psychological and behavioral disease during developmental age: the importance of the alliance with parents. *Neuropsychiatr. Dis. Treat.* 5 541–546. 10.2147/NDT.S5880 19898668 PMC2773285

[B31] Gillett K. S. Harper J. M. Larson J. H. Berrett M. E. Hardman R. K. (2009). Implicit family process rules in eating-disordered and non-eating-disordered families. *J. Marital Fam. Ther.* 35 159–174. 10.1111/j.1752-0606.2009.00113.x 19302514

[B32] Godart N. Berthoz S. Curt F. Perdereau F. Rein Z. Wallier J. (2012). A randomized controlled trial of adjunctive family therapy and treatment as usual following inpatient treatment for anorexia nervosa adolescents. *PLoS ONE* 7:e28249 10.1371/journal.pone.0028249 PMC3251571 22238574

[B33] Gottman J. (1997). *The Heart of Parenting.* New York, NY: Simon and Schuster.

[B34] Gowers S. North C. (1999). Difficulties in family functioning and adolescent anorexia nervosa. *Br. J. Psychiatry* 174 63–66. 10.1192/bjp.177.2.179 10211153

[B35] Hay P. Chinn D. Forbes D. Madden S. Newton R. Sugenor L. (2014). Royal Australian and New Zealand College of Psychiatrists clinical practice guidelines for the treatment of eating disorders. *Aust. N. Z. J. Psychiatry* 48 977–1008. 10.1177/0004867414555814 25351912

[B36] Hedenbro M. Rydelius P. A. (2014). Early interaction between infants and their parents predicts social competence at the age of four. *Acta Paediatr.* 103 268–274. 10.1111/apa.12512 24669379

[B37] Herpertz-Dahlmann B. Seitz J. Konrad K. (2011). Aetiology of anorexia nervosa: from a “psychosomatic family model” to a neuropsychiatric disorder? *Eur. Arch. Psychiatry Clin. Neurosci.* 261(Suppl. 2), S177–S181. 10.1007/s00406-011-0246-y 21866370

[B38] Herpertz-Dahlmann B. van Elburg A. Castro-Fornieles J. Schmidt U. (2015). ESCAP Expert Paper: new developments in the diagnosis and treatment of adolescent anorexia nervosa–a European perspective. *Eur. Child Adolesc. Psychiatry* 24 1153–1167. 10.1007/s00787-015-0748-7 26226918 PMC4592492

[B39] Herzog D. B. Nussbaum K. M. Marmor A. K. (1996). Comorbidity and outcome in eating disorders. *Psychiatr. Clin. N. Am.* 19 843–859. 10.1016/S0193-953X(05)70385-3 9045226

[B40] Hollingshead A. B. (1975). *Four Factor Index of Social Status.* New Haven, CT: Yale University, Department of Sociology.

[B41] Holtom-Viesel A. Allan S. (2014). A systematic review of the literature on family functioning across all eating disorder diagnoses in comparison to control families. *Clin. Psychol. Rev.* 34 29–43. 10.1016/j.cpr.2013.10.005 24321132

[B42] Horesh N. Sommerfeld E. Wolf M. Zubery E. Zalsman G. (2015). Father-daughter relationship and the severity of eating disorders. *Eur. Psychiatry* 30 114–120. 10.1016/j.eurpsy.2014.04.004 24908149

[B43] Kaufman J. Birmaher B. Brent D. A. Ryan N. D. Rao U. (2000). K-SADS-PL. *J. Am. Acad. Child. Adolesc. Psychiatry* 39:1208 10.1097/00004583-200010000-00002 11026169

[B44] Kitzmann K. M. (2000). Effects of marital conflict on subsequent triadic family interactions and parenting. *Dev. Psychol.* 36 3–13. 10.1037/0012-1649.36.1.3 10645740

[B45] Kooiman C. G. Van Rees Vellinga S. Spinhoven P. Draijer N. Trijsburg R. W. Rooijmans H. G. M. (2004). Childhood adversities as risk factors for alexithymia and other aspects of affect dysregulation in adulthood. *Psychother. Psychosom.* 73 107–116. 10.1159/000075542 14767153

[B46] Lamb M. E. (2010). *The Role of the Father in Child Development.* Hoboken, NJ: John Wiley and Sons.

[B47] Lavadera A. L. Laghi F. Togliatti M. M. (2011). Assessing family coordination in divorced families. *Am. J. Fam. Ther.* 39 277–291. 10.1080/01926187.2010.539479

[B48] Le Grange D. Eisler I. (2009). Family interventions in adolescent anorexia nervosa. *Child Adolesc. Psychiatr. Clin N. Am.* 18 159–173. 10.1016/j.chc.2008.07.004 19014864

[B49] Le Grange D. Lock J. Loeb K. Nicholls D. (2010). Academy for Eating Disorders position paper: the role of the family in eating disorders. *Int. J. Eat. Disord.* 43 1–5. 10.1002/eat.20751 19728372

[B50] Lock J. (2011). Evaluation of family treatment models for eating disorders. *Curr. Opin. Psychiatry* 24 274–279. 10.1097/YCO.0b013e328346f71e 21519263

[B51] Lock J. La Via M. C. American Academy of Child and Adolescent Psychiatry [AACAP] and Committee on Quality Issues [CQI] (2015). Practice parameter for the assessment and treatment of children and adolescents with eating disorders. *J. Am. Acad. Child Adolesc. Psychiatry* 54 412–425. 10.1016/j.jaac.2015.01.018 25901778

[B52] Lyke J. Matsen J. (2013). Family functioning and risk factors for disordered eating. *Eat. Behav.* 14 497–499. 10.1016/j.eatbeh.2013.08.009 24183144

[B53] Malagoli Togliatti M. Mazzoni S. (2006). *Osservare, Valutare e Sostenere la Relazione Genitori-figli. Il Lausanne Trilogue Play Clinico LTPc* Milano: Raffaello Cortina.

[B54] Mann C. J. (2003). Observational research methods. Research design II: cohort, cross sectional, and case-control studies. *Emerg. Med. J.* 20 54–60. 10.1136/emj.20.1.54 12533370 PMC1726024

[B55] Mannarini S. (2009). A method for the definition of self-awareness behavior dimension with clinical subjects: a latent trait analysis. *Behav. Res. Methods* 41 1029–1037. 10.3758/BRM.41.4.1029 19897811

[B56] Mannarini S. Balottin L. Munari C. Gatta M. (2016a). Assessing conflict management in the couple: the definition of a latent dimension. *Fam. J.* 10.1177/1066480716666066

[B57] Mannarini S. Balottin L. Toldo I. Gatta M. (2016b). Alexithymia and psychosocial problems among Italian preadolescents. A latent class analysis approach. *Scand. J. Psychol.* 57 473–481. 10.1111/sjop.12300 27376760

[B58] Mannarini S. Boffo M. (2014). The relevance of confidence and security: a latent domain of attachment relationships. *Scand. J. Psychol.* 55 53–59. 10.1111/sjop.12091 24303992

[B59] Mannarini S. Boffo M. (2015). Anxiety, bulimia, drug and alcohol addiction, depression, and schizophrenia: what do you think about their aetiology, dangerousness, social distance, and treatment? A latent class analysis approach. *Soc. Psychiatry Psychiatr. Epidemiol.* 50 27–37. 10.1007/s00127-014-0925-x 24972643

[B60] Mannarini S. Boffo M. Balottin L. (2013). Beliefs about the patient’s role in the psychotherapeutic relationship: a latent trait perspective. TPM. Test. *Psychom. Methodol. Appl. Psychol.* 20 277–294. 10.4473/TPM20.3.6

[B61] Manninen M. Therman S. Suvisaari J. Ebeling H. Moilanen I. Huttunen M. (2011). Alexithymia is common among adolescents with severe disruptive behavior. *J. Nerv. Ment. Dis.* 199 506–509. 10.1097/NMD.0b013e3182214281 21716065

[B62] Mazzoni S. Lavadera A. L. Di Benedetto R. Criscuolo M. Mangano C. (2015). Parenting coalitions: coparenting and toddler’s interactive styles. *Psicol. Clin. Dello Sviluppo* 19 79–100. 10.1449/79740

[B63] Mazzoni S. Lubrano Lavadera A. (2013). “Le jeu trilogique de lausanne (ltp) en clinique: application dans le contexte d’interventions de soutien à la relation parents-enfants. [The lausanne trilogue play (LTP) in the clinical context: application for the supportive interventions on the parent-child relationship],” in *Naitre et Grandir en Sein de la Triade: le Developpement de l’alliance Familiale [Borning and Growing up in the Triad: the Development of the Family Alliance*], eds Favez N. Frascarolo-Moutinot F. Tissot H. (Bruxelles: De Boeck), 177–192.

[B64] McDermott B. M. Batik M. Roberts L. Gibbon P. (2002). Parent and child report of family functioning in a clinical child and adolescent eating disorders sample. *Aust. N. Z. J. Psychiatry* 36 509–514. 10.1046/j.1440-1614.2002.01043.x 12169151

[B65] McHale J. Kuersten Hogan R. Lauretti A. (2001). “Evaluating coparenting and family level dynamics during infancy and early childhood: the coparenting and family rating system,” in *Family Observational Coding Systems*, eds Kerig P. Lindahl E. K. (Mahwah, NJ: Erlbaum).

[B66] McHale J. P. Coates E. E. (2014). Observed coparenting and triadic dynamics in African American fragile families at 3 months’ postpartum. *Infant Ment. Health J.* 35 435–451. 10.1002/imhj.21473 25798494

[B67] Minuchin S. Baker L. Rosman B. L. Liebman R. Milman L. Todd T. C. (1975). A conceptual model of psychosomatic illness in children: family organization and family therapy. *Arch. Gen. Psychiatry* 32 1031–1038. 10.1001/archpsyc.1975.01760260095008 808191

[B68] Murray S. B. Le Grange D. (2014). Family therapy for adolescent eating disorders: an update. *Curr. Psychiatry Rep.* 16:447 10.1007/s11920-014-0447-y 24652505

[B69] National Institute for Health, and Care Excellence [NIHCE] (2004). *Eating Disorders: Core Interventions in the Treatment and Management of Anorexia Nervosa, Bulimia Nervosa and Related Eating Disorders.* London: NICE. 23346610

[B70] Nicholls D. E. Viner R. M. (2009). Childhood risk factors for lifetime anorexia nervosa by age 30 years in a national birth cohort. *J. Am. Acad. Child Adolesc. Psychiatry* 48 791–799. 10.1097/CHI.0b013e3181ab8b75 19564797

[B71] Novick K. K. Novick J. (2011). *Working with Parents Makes Therapy Work.* Lanham, MD: Rowman and Littlefield Publishers.

[B72] Ravi S. Forsberg S. Fitzpatrick K. Lock J. (2009). Is there a relationship between parental self-reported psychopathology and symptom severity in adolescents with anorexia nervosa? *Eat. Disord.* 17 63–71. 10.1080/10640260802570122 19105061

[B73] Rieffe C. De Rooij M. (2012). The longitudinal relationship between emotion awareness and internalising symptoms during late childhood. *Eur. Child Adolesc. Psychiatry* 21 349–356. 10.1007/s00787-012-0267-8 22466448 PMC3370159

[B74] Rikani A. A. Choudhry Z. Choudhry A. M. Ikram H. Asghar M. W. Kajal D. (2013). A critique of the literature on etiology of eating disorders. *Ann. Neurosci.* 20 157–161. 10.5214/ans.0972.7531.200409 25206042 PMC4117136

[B75] Rodríguez Martín A. Novalbos Ruiz J. P. Martínez Nieto J. M. Escobar Jiménez L. Castro De Haro A. L. (2004). Epidemiological study of the influence of family andsocioeconomic status in disorders of eating behaviour. *Eur. J. Clin. Nutr.* 58 846–852. 10.1038/sj.ejcn.1601884 15164104

[B76] Schoppe-Sullivan S. J. Altenburger L. E. Settle T. A. Kamp Dush C. M. Sullivan J. M. Bower D. J. (2014). Expectant fathers’ intuitive parenting: associations with parent characteristics and postpartum positive engagement. *Infant Ment. Health J.* 35 409–421. 10.1002/imhj.21468 25798492 PMC4575558

[B77] Sim L. A. Homme J. H. Lteif A. N. Vande Voort J. L. Schak K. M. Ellingson J. (2009). Family functioning and maternal distress in adolescent girls with anorexia nervosa. *Int. J. Eat. Disord.* 42 531–539. 10.1002/eat.20654 19189407

[B78] Simonelli A. Bighin M. Palo F. (2012). Coparenting interactions observed by the prenatal lausanne trilogue play: an Italian replication study. *Infant Ment. Health J.* 33 609–619. 10.1002/imhj.21350 28520113

[B79] Steinhausen H. C. (2009). Outcome of eating disorders. *Child Adolesc. Psychiatr. Clin. N. Am.* 18 225–242. 10.1016/j.chc.2008.07.013 19014869

[B80] Sullivan P. F. (1995). Mortality in anorexia nervosa. *Am. J. Psychiatry* 152 1073–1074. 10.1176/ajp.152.7.1073 7793446

[B81] Swanson S. A. Crow S. J. Le Grange D. Swendsen J. Merikangas K. R. (2011). Prevalence and correlates of eating disorders in adolescents. Resultsfrom the national comorbidity survey replication adolescent supplement. *Arch. Gen. Psychiatry* 68 714–723. 10.1001/archgenpsychiatry.2011.22 21383252 PMC5546800

[B82] Taylor G. J. Bagby R. M. Parker J. D. A. (1997). *Disorder of Affect Regulation. Alexithymia in Medical and Psychiatric Illness.* Cambridge: Cambridge University Press.

[B83] Wallin U. Kronvall P. (2002). Anorexia nervosa in teenagers: change in family function after family therapy, at 2-year follow-up. *Nord. J. Psychiatry* 56 363–369. 10.1080/080394802760322132 12470310

[B84] Woodside D. B. Lackstrom J. Shekter-Wolfson L. Heinmaa M. (1996). Long-term follow-up of patient-reported family functioning in eating disorders after intensive day hospital treatment. *J. Psychosom. Res.* 41 269–277. 10.1016/0022-3999(96)00118-3 8910249

